# The Clinical Value of VDR and CTLA 4 in Evaluating the Prognosis of Invasive Duct Carcinoma of Egyptian Patients and their Benefit as a Target Therapy

**DOI:** 10.31557/APJCP.2021.22.4.1183

**Published:** 2021-04

**Authors:** Nanis Shawky Holah

**Affiliations:** *Department of Pathology, Faculty of Medicine, Menoufia University, Egypt. *

**Keywords:** Breast carcinoma, CTLA 4, Immunotherapy, Immunohistochemistry and VDR

## Abstract

**Objective:**

Breast cancer represents the second most common female malignancies worldwide and the most common in Egypt. The nuclear vitamin D receptor plays a role in the biology of cancer by affecting inflammatory microenvironment. The aim of this study is to evaluate the role of VDR and CTLA 4 in invasive duct carcinoma of Egyptian patients.

**Methods:**

This is a retrospective study that included 70 invasive duct carcinoma specimens retrieved from the archival material of Pathology Department, Faculty of medicine, Menoufia University, Egypt, spanning the period between January 2010 and December 2017. All cases were stained for VDR and CTLA 4 antibodies.

**Results:**

There is significant association between high *VDR* expression in tumor cells and parameters of good prognosis as low tumor stage (T1) and (N0) stage. On the other hand, there is significant association between low *CTLA4* tumor expression and good prognostic parameters as low tumor stage (T1) and absent vascular invasion. Regarding lymphocyte expression, there is significant association between positive *CTLA4* expression in lymphocytes and parameters of good prognosis as absent metastasis. High *VDR *tumor expression is the most independent prognostic factor on overall survival of breast carcinoma patients.

**Conclusion:**

high *VDR* expression in tumor cells is associated with good prognostic parameters and is the most independent prognostic factor on overall survival so it might be of benefit as a target therapy for Egyptian invasive duct carcinoma patients and VDR might augment the expression of *CTLA-4*, So tailored immunotherapy might have an impact on invasive duct carcinoma patients.

## Introduction

Breast cancer represents 24.5% and is considered the first most common female malignancies worldwide (GLOBOCAN, 2020), while in Egypt it represents 38.8% and is considered the most common female cancer and the second cause of female cancer death after lung cancer (Ibrahim et al., 2014; El Bolkainy et al., 2016).

The source of vitamin D in the body may be endogenous through sun exposure (up to 90%) or exogenous through dietary and supplemental intake (up to 10%) (Holick, 2006). There are many controversies between the relation between vitamin D and reduced breast cancer risk ( Robien et al., 2007; Wang et al., 2014).

The active form of vitamin D (1,25(OH)2D) binds to the vitamin D receptor (VDR), a ligand-dependent transcription factor, that regulates transcription of a number of genes involved in apoptosis, growth factor signaling, cell proliferation, differentiation and immunomodulation (McCullough et al., 2007). Presence of VDR in normal breast epithelial cells and breast cancer cells suggests a relation between it and breast cancer risk (Zhang and Song, 2014).

Other studies found that it inhibits breast cancer cell line growth (Murray et al., 2017) and induce autophagy in breast cancer cell line (Tavera-Mendoza et al., 2017). 

There is an important role for VDR in modulating the inflammation system by regulating the production of inflammatory cytokines and immune cells, which are important for the pathogenesis of many immune-related diseases (Wei et al., 2018).

Cytotoxic T lymphocyte associated protein-4 (CTLA-4) (CD152) is one of inhibitory immune checkpoints that have been identified to suppress anti-tumor immune responses in solid tumors. It is a CD28 homolog and primarily located in intracellular compartments in resting naive T cells. This negative co-stimulatory molecule counteracts the activity of the positive co-stimulatory molecule CD28. Also *CTLA4 *expression was dyaregulated in papillary thyroid carcinoma (Tuccilli et al., 2018). And there is discrepancy whether *CTLA-4 *expression in tumor cells has a good or a bad prognostic impact (Xu et al., 2018). 

Breast cancer is the most common malignancy in Egyptian females and despite the advances in surgery and diagnosis, the prognosis and survival of the patients are still unsatisfactory, so it is mandatory to search for different parameters related to prognosis and therapy. Also studies of tumor cells showed the important regulatory effect of vitamin D on the inflammatory microenvironment, but the evidence linking vitamin D and immune response in cancer is still scarce (Liu et al., 2018). The aim of this study is to evaluate the immunohistochemical expression of *VDR* and CTLA4 in invasive duct carcinoma of Egyptian patients and their relation to the available clinicopathological data.

## Materials and Methods

This retrospective study was conducted on 70 breast invasive duct carcinoma cases obtained from the archival cases of Pathology Department, faculty of medicine, Menoufia University, in the period between January 2010 and December 2017. Clinical and survival data were retrieved from medical patients’ files. 

Inclusion criteria:

1- The type of breast carcinoma: we selected only invasive duct carcinoma not otherwise specified type

2- The type of surgery: we selected radical mastectomy specimens only.

3- Therapy: cases that didn’t receive any neoadjuvant therapy received 

Exclusion criteria: 

1- The type of breast carcinoma: we exclude any type other than invasive duct carcinoma not otherwise specified type

2- The type of surgery: we excluded core, incision and excision biopsies.

3- Therapy: we exclude any case that received any neoadjuvant therapy.

*Histopathological Evaluation*

From each representative paraffin block, 4-μm thick serial sections were cut and stained with haematoxylin and eosin stain.

The clinicopathological data as histological grade, hormonal status, vascular, perineural invasion, extracapsular nodal invasion, Multicentricity and metastasis were obtained from the patients’ sheets and TNM staging system (2010) is used for staging of the tumor according to size into T1, T2, T3 and T4 and to nodal status into Nx, N0, N1, N2 and N3 (Edge, 2010).

*Immunohistochemical Staining*

About 4-μm thick sections of these blocks were cut and mounted on positive charged slides and used for analysis by immunohistochemical method (streptavidin-biotin amplified system). As the paraffin-embedded tissue sections were deparaffinized in xylene andrehydrated. The sections were treated with 10mMcitrate buffer, pH 6.0, at 961C for 10 to 20min, followed by 10 mL of Tris-EDTA for 10 to 20 min. Endogenous peroxidase was blocked with peroxidase-blocking reagent (cat. #TP-015-HD) (Lab Vision Cooperation, Fremont, CA) using VDR (concentrated mouse monoclonal antibody, clone 9A7, Catalog # MA1-710, Thermo Fesher sientific CA, a dilution of 1:100), CTLA4 (concentrated polyclonal rabbit anti-human anti CD152, United States Biological, a dilution of 1:50), HER2 rabbit monoclonal antibody (c-erbB-2), clone GR011, ready to use, Genemed Biotechnologies, Inc., ER (concentrated rabbit monoclonal antibody, clone SP1, Cat #MA5-14501, Thermo Fesher sientific CA, a dilution of 1:200) and PR (concentrated mouse monoclonal antibody, clone Alpha PR6, Catalog # MA1-411, Thermo Fesher sientific CA, a dilution of 1:100).

A positive reaction was revealed using the streptavidinbiotin- peroxidase technique (cat. #TP-015-HD) (Lab Vision Cooperation) with chromogen DAB. The sections were then counterstained with Mayer’s haematoxylin (cat. No. 94583; Bio Genex) for 30 to 60 s to stain nuclei. Sections were washed in tap water for 5 min. Placental tissue and human tonsil tissue were used as positive controls for VDR and CTLA4 respectively and normal breast tissue was used as positive control for ER, PR and HER2 neu.

*Interpretation of VDR and CTLA-4 immunostaining*

Expression: *VDR* was assessed in tumor cells and positive expression was considered if any tumor cells showed positive brownish nuclear staining in any number of cells. While CTLA4 was assessed in tumor infiltrating lymphocytes as well as tumor cells. Positive expression was considered if any tumor cells and/or lymphocytes showed positive brownish cytoplasmic staining in any number of cells (Adisa et al., 2017; Chang et al., 2017).

Intensity: In both markers the staining intensity was also reported and scored from 0 to 3 (0=Negative, 1=Mild staining, 2=Moderate staining and 3=Strong staining) for each cells (Adisa et al., 2017; Chang et al., 2017).

Percentage: In both markers the percentage of positive cells was counted. The median percentage of positive cells was used as a cutoff point and the cases were divided into two groups: 

• Low percentage: ≤ the median 

• High percentage: > the median (Chang et al., 2017).

*Interpretation of ER and PR Immunostaining*

Bothe ER and PR showed nuclear staining and we assess the percentage and the intensity of the tumor cell, regarding percentage, both ER and PR are considered positive when more than 1% cells showed nuclear staining. The intensity of the staining was graded as weak, moderate and strong. It was considered negative when internal control cells present showed no nuclear staining (Longacre et al., 2017).

*Interpretation of Her 2 neu Immunostaining*

Only membranous staining was considered and Her 2 neu positivity was assessed using the following scoring system: (Yan et al., 2014). 

0 : No membrane staining or less than 10% of cells.

1+: Partial membrane staining in more than 10% of cells.

2+: Weak, circumferential membrane staining in more than 10% of cells, or intense membrane staining in less than 30% of cells.

3+: Intense membrane staining in more than 30% of cells.

*Score 0,1&2 were considered negative for HER2 and score 3 was considered positive for HER2.

*Overall survival data*

Overall survival time is the length of time from either the date of diagnosis or the start of treatment for a disease, such as cancer, that patients diagnosed with the disease are still alive. In a clinical trial, measuring the overall survival is one way to see how well a new treatment works.

By revision of patients’ files for breast carcinoma cases ranged from 2010 to 2017, overall survival time was available for 68 out of 70 patients. 

*Statistical analysis*

The statistical analysis was conducted using SPSS “Statistical Package for the Social Sciences″ program for windows, version 20, SPSS Inc., Chicago, Illinois, USA. Mann-Whitney U and Kruskal-Wallis tests were used to compare nonparametric data, the chi-square test was used to assess the association between the clinicopathologic parameters and *VDR* and *CTLA 4 *expression and Kaplan–Meier test was used for survival analysis. P≤0.05 was considered to indicate statistical significance in all tests.

## Results

*Clinicopathological data of the studied invasive duct carcinoma are shown in* Table 1

The age of the studied breast carcinoma cases was ranged from 27-83 year with a median of 48 and mean ± SD of 48.228 ± 12.53.

**Plate1:**
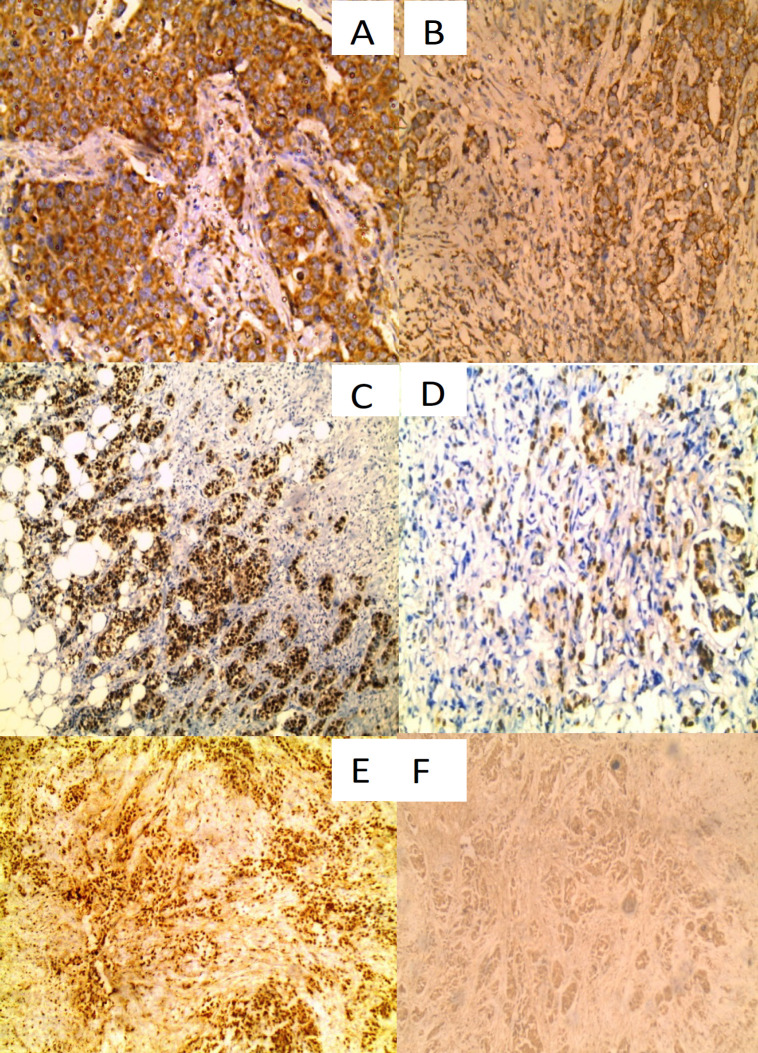
Immunohistochemical Staining of VDR and CTLA 4 in Breast Carcinoma Cases A: A case of IDC grade III exhibiting high cytoplasmic expression of CTLA 4 in tumor cells and positive expression in lymphocytes (IHC x 200), B: A case of IDC grade II exhibiting low cytoplasmic expression of CTLA 4 in tumor cells and positive expression in lymphocytes (IHC x 200), C: A case of IDC grade II exhibiting high nuclear expression of VDR (IHC x 200), D: A case of IDC grade II exhibiting low nuclear expression of VDR (IHC x 200). E: A case of IDC grade III exhibiting high nuclear expression of VDR (IHC x 200). F: A case of IDC grade I exhibiting low cytoplasmic expression of CTLA4 (IHC x 200).

The Immunohistochemical results of VDR and CTLA4 antibodies are shown in Table 2 and plate 1

The relationship of *VDR* tumor expression in the studied invasive duct carcinoma cases and the clinicopathological parameters:

There is significant association between high *VDR *tumor expression and good prognostic parameters as low tumor stage (T1) and (N0) nodal stage (P value= 0.02 and 0.01 respectively). Moreover, there is a trend of significance between positive VDR in the tumor cells and absent vascular invasion and absent metastasis (P value= 0.06 and 0.09 respectively). Also between high *VDR* expression in tumor cells and low grade (P= 0.09) (Table 3 and 4).

The relationship of *CTLA4* tumor and lymphocyte expression in the studied invasive duct carcinoma cases and the clinicopathological parameters:

There is significant association between positive *CTLA4* expression in lymphocytes and good prognostic parameters as absent metastasis (P= 0.018), and a trend of significance with low tumor stage (P= 0.08) (Table 5)

On the other hand there is significant association between high *CTLA4* expression in tumor cells and poor prognostic parameters as advanced tumor stage (T4) and presence of vascular invasion (P= 0.01 and 0.05 respectively), also a trend of significance with advanced nodal stage (N2 and N3) (P= 0.07) (Table 6).

The relationship of *VDR* and* CTLA4* expression in lymphocytes and tumor cells of the studied invasive duct carcinoma cases :

There is a direct association between positive *VDR* expression in tumor cells and positive and high *CTLA4* expression in lymphocytes (P= 0.000 and 0.09 respectively) (Table 7).

On the other hand there is inverse association between positive *VDR* expression in tumor cells and negative and low *CTLA4* expression in tumor cells (P= 0.03 and 0.01 respectively) (Table 8).

Furthermore, there is inverse association between positive and high *CTLA4* expression in tumor cells and negative and low expression in lymphocytes (P= 0.001 and 0.000 respectively for positivity and 0.005 and 0.009 respectively for high score of expression) (Table 9).

*Overall survival data*

By revising the files for breast carcinoma patients from 2010 to 2017, overall survival time was available for 97% of patients and with the range of 1 to 121 months, mean ± standard deviation of 44.94 ± 35.953 and a median of 26.5 months.

*Univariate survival analysis for breast carcinoma cases*

**Figure 1:**
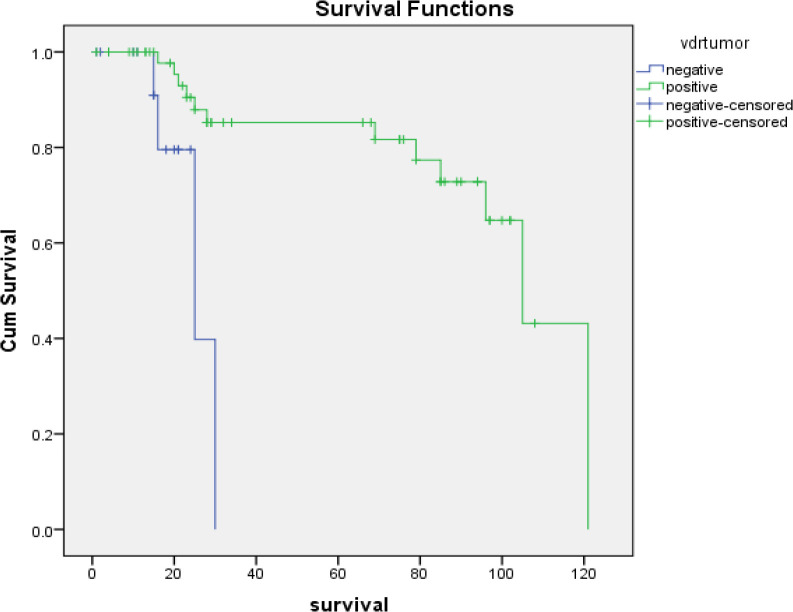
Kaplan-Meier Overall Survival for Breast Carcinoma Patients with Positive or Negative VDR Tumor Expression (P=0.001 ''highly significant'', Log-Rank=10.501)

**Figure 2:**
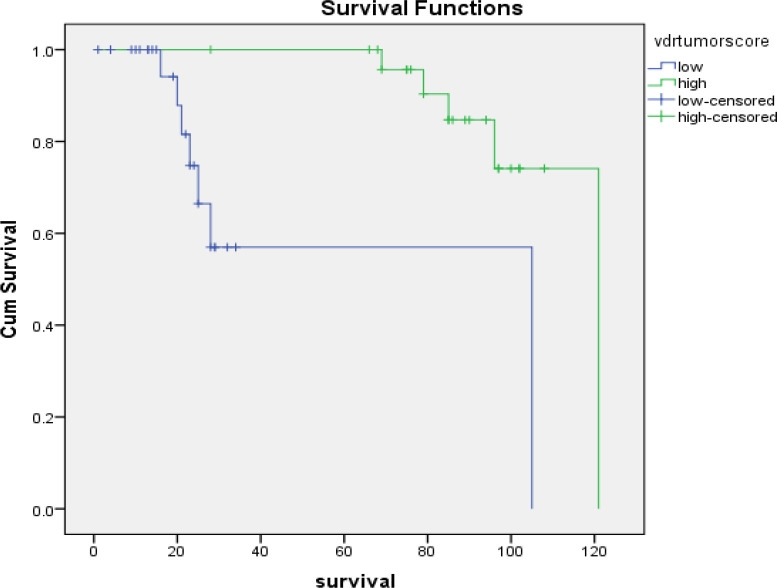
Kaplan-Meier Overall Survival for Breast Carcinoma Patients with Low or High *VDR* Tumor Score of Expression (P=0.001 ''highly significant'', Log-Rank=11.677).

Univariate survival analysis revealed that positive *VDR* tumor expression (P-value = 0.001) and high VDR tumor expression (p-value = 0.001) have a good prognostic impact on the outcome of patients, but CTLA4 and other variables weren’t significant (Figures 1 and 2).

*Multivariate survival analysis for breast carcinoma cases*

**Figure 3:**
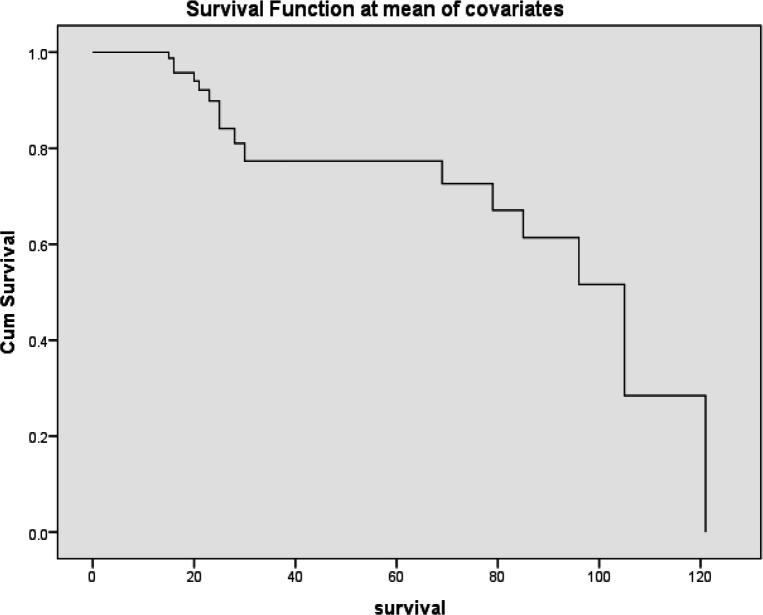
Cox Regression Survival Curve Showing *VDR* Tumor Expression in Breast Carcinoma Patients

**Figure 4:**
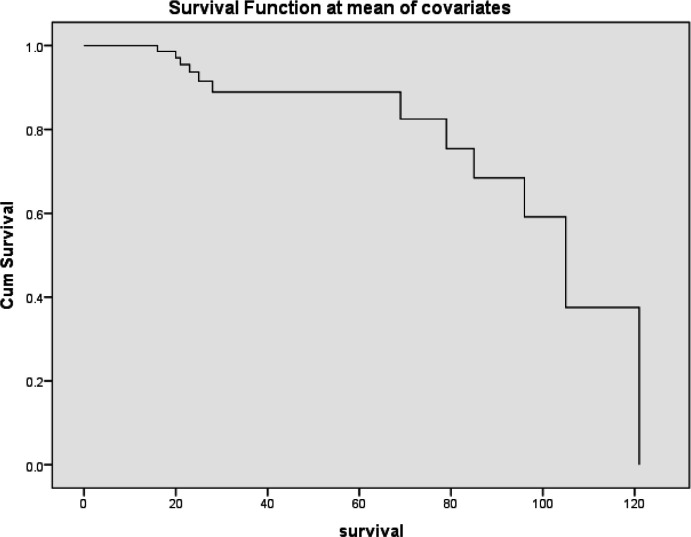
Cox Regression Survival Curve Showing VDR Tumor Score of Expression in Breast Carcinoma Patients

From multivariate survival analysis, high VDR tumor expression was proved to be the most and the first independent prognostic factor on overall survival of breast carcinoma patients i.e VDR is a favorable prognostic indicator for breast carcinoma. (Table 10) (Figure 3and 4)

**Table 1:** Clinicopathological Data of the Studied Invasive Duct Carcinoma Cases

Variable	Number (percent)
Tumor stage:	
T1	5 (7.1)
T2	12 (17.1)
T3	44 (62.9)
T4	9 (12.9)
Nodal stage :	
Nx	15 (21.4)
N0	14 (20)
N1	8 (11.4)
N2	15 (21.4)
N3	18 (25.7)
Multicentricity	
Present	9 (12.9)
Absent	61 (87.1)
Vascular invasion:	
Present	5 (7.1)
Absent	65 (92.9)
Perineural invasion	
Present	3 (4.3)
Absent	67 (95.7)
Hormonal status:	
ER+, PR+ and Her2 neu+	10 (14.3)
ER-, PR- and Her2 neu+	8 (11.4)
Triple negative	15 (21.4)
ER+, PR+ and Her2 neu-	27 (38.6)
ER+, PR- and Her2 neu-	6 (8.6)
ER-, PR+ and Her2 neu+	0 (0)
ER-, PR+ and Her2 neu-	0 (0)
ER+, PR- and Her2 neu+	4 (5.7)
Her2 neu status:	
Positive	24 (34.3)
Negative	44 (62.9)
Equivocal	2 (2.9)
Grade	
I	6 (8.6)
II	18 (25.7)
III	46 (65.7)
Extracapsular nodal invasion:	
Present	4 (5.7)
Absent	66 (94.3)
Metastasis	
Present	9 (12.9)
Absent	61 (87.1)

SD, Standard deviation; M: F, Male to female; Tumor and nodal staging according to AJCC Cancer Staging Manual, 7^th^ ed

**Table 2:** Immunohistochemical Results of *VDR* and *CTLA4* Antibodies in the Studied Invasive Duct Carcinoma Cases

Variables	Number (percent)
*VDR* expression in tumor cells:	
Positive	55 (78.6)
Negative	15 (21.4)
*VDR* intensity of expression in tumor cells: (55 cases)	
Mild	23 (41.8)
Moderate	14 (25.5)
Strong	18 (32.7)
*VDR* score of expression in tumor cells: (55 cases)	
Low	29 (52.7)
High	26 (47.3)
*CTLA4* expression in lymphocytes:	
Positive	42 (60)
Negative	28 (40)
*CTLA4* score of expression in lymphocytes:(42 cases)	
Low	20 (47.6)
High	22 (52.4)
*CTLA4* expression in tumor:	
Positive	57 (81.4)
Negative	13 (18.6)
*CTLA4* score of expression in tumor:(57 cases)	
Low	27 (47.4)
High	30 (52.6)

**Table 3:** The Relationship of *VDR* Tumor Expression in the Studied Invasive Duct Carcinoma Cases and the Clinicopathological Parameters

Variables	*VDR* tumor expression		P value
	Negative Number (percent)	Positive Number (Percent)	
Tumor stage			0.141
T1	0 (0)	5 (100 )	
T2	1 (8.3)	11 (91.7)	
T3	10 (22.7)	34 (77.3)	
T4	4 (44.4)	5 (55.6)	
Nodal stage:			0.559
Nx	4 (26.7)	11 (73.3)	
N0	1 (7.1)	13 (92.9)	
N1	1 (12.5)	7 (87.5)	
N2	4 (26.7)	11 (73.3)	
N3	5 (27.8)	13 (72.2)	
Multicentricity			0.379
Present	1 (11.1)	8 (88.9)	
Absent	14 (23)	47 (77)	
Vascular invasion:			0.06
Present	3 (60)	2 (40)	
Absent	12 (18.5)	53 (81.5)	
Perineural invasion:			0.521
Present	1 (33.3)	2 (66.7)	
Absent	14 (20.9)	53 (79.1)	
Hormonal status:			0.735
ER+, PR+ and Her2 neu+	1 (10)	9 (90)	
ER-, PR- and Her2 neu+	2 (25)	6 (75)	
Triple negative	3 (20)	12 (80)	
ER+, PR+ and Her2 neu-	7 (25.9)	20 (74.1)	
ER+, PR- and Her2 neu-	2 (33.3)	4 (66.7)	
ER+, PR- and Her2 neu+	0 (0)	4 (100)	
Her2 neu			0.276
Positive	3 (12.5)	21 (87.5)	
Negative	12 (27.3)	32 (72.7)	
Equivocal	0 (0)	2 (100)	
Grade			0.132
I	0 (0)	6 (100)	
II	2 (11.1)	16 (88.9)	
III	13 (28.3)	33 (71.7)	
Extracapsular nodal invasion:			0.628
Present	1 (25)	3 (75)	
Absent	14 (21.2)	52 (78.8)	
Metastasis			0.09
Present	4 (44.4)	5 (55.6)	
Absent	11 (18)	50 (82)	

SD, Standard deviation; X^2^, Chi square

**Table 4:** The Relationship of *VDR* Tumor Score of Expression in the Studied Invasive Duct Carcinoma Cases and the Clinicopathological Parameters

Variables	*VDR* tumor score of expression		P value
	Low Number (percent)	High Number (percent)	
Tumor stage			0.02*
T1	0 (0 )	5 (100 )	
T2	4 (36.4)	7 (63.6)	
T3	21 (61.8)	13 (38.2)	
T4	4 (80)	1 (20)	
Nodal stage:			0.01*
Nx	10 (90.9)	1 (9.1)	
N0	4 (30.8)	9 (69.2)	
N1	2 (28.6)	5 (71.4)	
N2	4 (36.4)	7 (63.6)	
N3	9 (69.2)	4 (30.8)	
Multicentricity			0.417
Present	5 (62.5)	3 (37.5)	
Absent	24 (51.1)	23 (48.9 )	
Vascular invasion:			0.727
Present	1 (50)	1 (50)	
Absent	28 (52.8)	25 (47.2)	
Perineural invasion:			0.727
Present	1 (50)	1 (50)	
Absent	28 (52.8)	25 (47.2)	
Hormonal status:			0.421
ER+, PR+ and Her2 neu+	5 (55.6)	4 (44.4)	
ER-, PR- and Her2 neu+	5 (83.3)	1 (16.7)	
Triple negative	7 (58.3)	5 (41.7)	
ER+, PR+ and Her2 neu-	10 (50)	10 (50)	
ER+, PR- and Her2 neu-	1 (25)	3 (75)	
ER+, PR- and Her2 neu+	1 (25)	3 (75)	
Her2 neu			0.302
Positive	12 (57.1)	9 (42.9)	
Negative	17 (53.1)	15 (46.9)	
Equivocal	0 (0)	2 (100)	
Grade			0.09
I	1 (16.7)	5 (83.3)	
II	11 (68.8)	5 (31.3)	
III	17 (51.5)	16 (48.5)	
Extracapsular nodal invasion:			0.542
Present	2 (66.7)	1 (33.3)	
Absent	27 (51.9)	25 (48.1)	
Metastasis:			0.212
Present	4 (80)	1 (20)	
Absent	25 (50)	25 (50)	

SD, Standard deviation; X^2^, Chi square; *, Significant

**Table 5:** The Relationship of *CTLA4* Lymphocyte Expression in Invasive Duct Carcinoma Cases and the Studied Clinicopathological Parameters

Variables	*CTLA4* lymphocyte expression		P value
	Negative Number (percent)	Positive Number (percent)	
Tumor stage			0.08
T1	1 (20 )	4 (80 )	
T2	4 (33.3)	8 (66.7)	
T3	16 (36.4)	28 (63.6)	
T4	7 (77.8)	2 (22.2)	
Nodal stage:			0.421
Nx	8 (53.3)	7 (46.7)	
N0	5 (35.7)	9 (64.3)	
N1	1 (12.5)	7 (87.5)	
N2	6 (40)	9 (60)	
N3	8 (44.4)	10 (55.6)	
Multicentricity			0.214
Present	2 (22.2)	7 (77.8)	
Absent	26 (42.6)	35 (57.4)	
Vascular invasion:			0.312
Present	3 (60)	2 (40)	
Absent	25 (38.5)	40 (61.5)	
Perineural invasion:			0.356
Present	2 (66.7)	1 (33.3)	
Absent	26 (38.8)	41 (61.2)	
Hormonal status:			0.46
ER+, PR+ and Her2 neu+	3 (30)	7 (70)	
ER-, PR- and Her2 neu+	2 (25)	6 (75)	
Triple negative	5 (33.3)	10 (66.7)	
ER+, PR+ and Her2 neu-	15 (55.6)	12 (44.4)	
ER+, PR- and Her2 neu-	2 (33.3)	4 (66.7)	
ER+, PR- and Her2 neu+	1 (25)	3 (75)	
Her2 neu			0.406
Positive	7 (29.2)	17 (70.8)	
Negative	20 (45.5)	24 (54.5)	
Equivocal	1 (50)	1 (50)	
Grade			0.449
I	1 (16.7)	5 (83.3)	
II	7 (38.9)	11 (61.1)	
III	20 (43.5)	26 (56.5)	
Extracapsular nodal invasion:			0.473
Present	1 (25)	3 (75)	
Absent	27 (40.9)	39 (59.1)	
Metastasis			0.018*
Present	7 (77.8)	2 (22.2)	
Absent	21 (34.4)	40 (65.6)	

SD, Standard deviation; X^2^, Chi square; *, Significant

**Table 6:** The Relationship of CTLA4 Tumor Score of Expression in Invasive Duct Carcinoma Cases and the Studied Clinicopathological Parameters

Variables	CTLA4 tumor score of expression		P value
	Low Number (percent)	High Number (percent)	
Tumor stage			0.01*
T1	2 (66.7 )	1 (33.3 )	
T2	9 (90 )	1 (10 )	
T3	13 (37.1)	22 (62.9)	
T4	3 (33.3)	6 (66.7)	
Nodal stage:			0.07
Nx	4 (33.3)	8 (66.7)	
N0	9 (81.8)	2 (18.2)	
N1	3 (50)	3 (50)	
N2	3 (25)	9 (75)	
N3	8 (50)	8 (50)	
Multicentricity			0.561
Present	3 (42.9)	4 (57.1)	
Absent	24 (48)	26 (52)	
Vascular invasion:			0.05*
Present	0 (0)	5 (100)	
Absent	27 (51.9)	25 (48.1)	
Perineural invasion:			0.139
Present	0 (0 )	3 (100 )	
Absent	27 (50)	27 (50 )	
Hormonal status:			0.119
ER+, PR+ and Her2 neu+	4 (57.1)	3 (42.9)	
ER-, PR- and Her2 neu+	3 (42.9)	4 (57.1)	
Triple negative	8 (61.5)	5 (38.5)	
ER+, PR+ and Her2 neu-	7 (31.8)	15 (68.2)	
ER+, PR- and Her2 neu-	1 (25)	3 (75)	
ER+, PR- and Her2 neu+	4 (100)	0 (0)	
Her2 neu			0.684
Positive	11 (55)	9 (45)	
Negative	15 (42.9)	20 (57.1)	
Equivocal	1 (50)	1 (50)	
Grade			0.516
I	3 (75)	1 (25)	
II	6 (46.2)	7 (53.8)	
III	18 (45)	22 (55)	
Extracapsular nodal invasion:			0.653
Present	2 (50)	2 (50)	
Absent	25 (47.2)	28 (52.8)	
Metastasis:			0.292
Present	3 (33.3)	6 (66.7)	
Absent	24 (50)	24 (50)	

SD, Standard deviation; X^2^, Chi square; *, Significant

**Table 7:** The Relationship of VDR and CTLA4 Expression in Lymphocytes and Tumor Cells of the Studied Invasive Duct Carcinoma Cases

Variables	VDR tumor expression		P value
	Positive Number (percent)	Negative Number (percent)	
CTLA4 lymphocyte expression:			0.000**
Negative	16 (57.1)	12 (42.9)	
Positive	39 (92.9)	3 (7.1)	
CTLA4 lymphocyte score of expression:			0.09
Low	17 (85)	3 (15)	
High	22 (100)	0 (0)	
CTLA4 tumor expression:			0.03*
Negative	13 (100)	0 (0)	
Positive	42 (73.7)	15 (26.3)	
CTLA4 tumor score of expression:			0.01*
Low	24 (88.9)	3 (11.1)	
High	18 (60)	12 (40)	

X^2^, Chi square; *, Significant; **, Highly significant

**Table 8:** The Relationship of VDR and CTLA4 Expression in Lymphocytes and Tumor Cells of the Studied Invasive Duct Carcinoma Cases

Variables	CTLA4 tumor expression		P value
	Negative Number (percent)	Positive Number (percent)	
CTLA4 lymphocyte expression:			0.001**
Negative	0 (0)	28 (100)	
Positive	13 (31)	29 (69)	
CTLA4 lymphocyte score of expression:			0.000**
Low	0 (0)	20 (100)	
High	13 (59.1)	9 (40.9)	
VDR tumor expression:			0.03*
Negative	0 (0)	15 (100)	
Positive	13 (23.6)	42 (76.4)	
VDR tumor score of expression:			0.41
Low	6 (20.7)	33 (79.3)	
High	7 (26.9)	19 (73.1)	

X^2^, Chi square; *, Significant; **, Highly significant

**Table 9:** The Relationship of VDR and CTLA4 Expression in Lymphocytes and Tumor Cells of the Studied Invasive Duct Carcinoma Cases

Variables	CTLA4 tumor score of expression		P value
	Low Number (percent)	High Number (percent)	
CTLA4 lymphocyte expression:			0.005**
Negative	8 (28.6)	20 (71.4)	
Positive	19 (65.5)	10 (34.5)	
CTLA4 lymphocyte score of expression:			0.009**
Low	10 (50)	10 (50)	
High	9 (100)	0 (0)	
VDR tumor expression:			0.01*
Negative	3 (20)	12 (80)	
Positive	24 (57.1)	18 (42.9)	
VDR tumor intensity of expression:			0.000**
Mild	5 (21.7)	18 (78.3)	
Moderate	12 (100)	0 (0)	
Strong	7 (100)	0 (0)	
VDR tumor score of expression:			0.344
Low	12 (52.2)	11 (47.8)	
High	12 (63.2)	7 (36.8)	

X^2^, Chi square; *, Significant; **, Highly significant

**Table 10:** Multivariate Cox Regression Analysis for Detection of the Independent Factors Affecting Patients’ Overall Survival

Variables	Exp(B)	CI 95 of Exp(B)		P-value
		Lower	upper	
VDR tumor expression	0.146	0.038	0.553	0.005**
VDR tumor score of expression	0.118	0.3	0.466	0.002**

CI, Confidence interval; **, highly significant

## Discussion

Breast cancer is the most common malignancy in Egyptian females and despite the advances in surgery and diagnosis, the prognosis and survival of the patients are still unsatisfactory, so it is mandatory to search for different parameters related to prognosis and therapy (GLOBOCAN, 2020) .

In this study we tried to evaluate the Immunohistochemical expression of *VDR* and* CTLA4* antidodies and their role in breast cancer of Egyptian patients as there is a great controversy about their role in suppression or promotion of breast cancer (Zhang et al., 2014; Murray et al., 2017; Tavera-Mendoza et al., 2017; Xu et al., 2018).

In the present study 58.2% of the studied cases showed moderate and strong *VDR* expression in tumor cells and this comes in line with Al-Azhri et al., (2016) who found 58% of the cases showed moderate and strong expression. On the other hand Ditsch et al., (2012) detected VDR positivity in 92% and Huss et al., (2019) who found nuclear positivity of VDR in 94.4% of the studied cases and Elsamany et al., (2020) who observed VDR positivity in 89.4% of the studied non metastatic breast cancer patients. These differences may be due to different techniques used in both studies and different number of the studied cases. 

In the current study there is significant association between high *VDR* tumor expression and parameters of good prognosis as low tumor stage (T1) and (N0) nodal stage, Moreover, there is a trend of significant between positive VDR in the tumor cells and absent vascular invasion and metastasis. Also between high *VDR* expression in tumor cells and low grade and these results come in line with Huss et al., (2019) who found that high expression of *VDR* is associated with favorable prognosis and low death rate in invasive duct carcinoma cases.

These results may be explained by the inhibitory intranuclear action of VDR as it inhibits breast cancer cell line growth (Murray et al., 2017) and induce autophagy in breast cancer cell line (Tavera-Mendoza et al., 2017). Also these results agreed with Zheng et al., (2017) who detected that *VDR* overexpression is associated with good prognostic parameters as small tumor size and low nodal stage and results may be explained as VDR inhibits breast cancer growth as it has pro-apoptotic and anti-proliferative effects through inhibition of the Wnt/β-catenin signaling pathway. Also regarding lung cancer, Increased *VDR* expression in lung adenocarcinoma is associated with improved survival. This may relate to a lower proliferative status and G1 arrest in high VDR-expressing tumors (Kim et al., 2012). Moreover, regarding prostate cancer, high *VDR* expression is also associated with good prognostic parameters as lower Gleason score and early tumor stage (Hendrickson et al., 2011)

On contrary to the present study El-Shorbagy et al., (2017) found that no association between breast cancer and VDR BsmI pleomorphism also other studies come in line with this opposite opinion (Chen et al., 2005; Wang et al., 2013; Zhang et al., 2014). These differences might be explained by different techniques used in different studies.

Regarding survival VDR is a favorable prognostic indicator for breast carcinoma and this come in line with Huss et al., (2019) but was opposite to that of Al-Azhri et al., (2016). 

From the present study as high VDR is good prognostic factor for breast carcinoma as it associated with low tumor and nodal stage together with low grade, absent vascular invasion and metastasis. So It may be used to personalize the adjuvant therapy used in treatment of breast carcinoma as Mastectomies were performed more often on VDR-negative tumors (55%) compared to VDR-positive tumors (41%). The postoperative treatment conference recommended adjuvant endocrine therapy for a smaller proportion and chemotherapy for a larger proportion of patients with VDR-negative tumors compared to VDR-positive tumors (Tavera-Mendoza et al., 2017). Also, vitamin d may be used to decrease the risk of breast carcinoma in Egyptian patients.

In the past, CTLA-4 has been found to be limited to T cells but nowadays, evidence reveals CTLA-4 expression in tumor cells. CTLA-4 is also expressed in non-lymphoid cells including placental fibroblasts, muscle cells and monocytes, suggesting that this molecule might be involved in controlling functions other than the widely described T-cell response inactivation (Wang et al., 2002). Surface expression of CTLA-4 is detectable by reverse transcriptase- PCR in all cell lines derived from a variety of human malignant solid tumors including carcinoma, melanoma, neuroblastoma, rhabdomyosarcoma and osteosarcoma (Contardi et al., 2005)

In the present study 40% of the lymphocytes and 81.4% of the studied invasive duct carcinoma cases show CTLA 4 positivity and these results are near those of Lan et al., (2018) regarding lymphocyte expression but not tumor expression as they found positive CTLA 4 in 41.2% of tumor and 46.1% of lymphocytes. Also, these results are in contrary with those of Mao et al., (2010) who found CTLA4 expression in 55% of lymphocytes and 100% of the studied breast carcinoma cases. These differences may be explained as the current study assessed CTLA4 expression only through semiquantitative immunohistochemistry, which may be less reliable than the other techniques used in different studies and due to heterogeneous study samples and different cut-off values of the CTLA-4 expression.

The present study showed that CTLA4 tumor expression was associated with poor prognostic parameters as there is significant association between low CTLA4 expression in tumor cells and low tumor stage (T1) and absent vascular invasion, also a trend of significance with (N0) nodal stage and these results come in line with Yu et al., (2015) and Wang et al., (2007) who found that the CTLA-4 gene may be associated with the progression of breast cancer in the Chinese Han population.

Also, some studies indicate that breast cancer patients with higher CTLA-4 mRNA levels had obvious axillary lymph node metastases and a higher clinical stage. Patients with high tumor CTLA-4 expression in mesothelioma, nasopharyngeal carcinoma and melanoma had a poorer prognosis than those with low expression, which suggested CTLA-4 as a potential target for tumor immunotherapy (Salvi et al., 2012; Huang et al., 2016; Roncella et al., 2016).

This inhibitory effect might potentially occur through the interaction between CTLA-4 expressed by the tumor cells and B7 ligands expressed by the tumor microenvironment cells including the antigen presenting cells (APC), such as dendritic cells (DCs), or antitumor activated T cells. This interaction might result in delivering CTLA-4-mediated negative signals into tumor cells leading to inhibition of their proliferation rate and/or induction of apoptotic cell death (Contardi et al., 3005) Also, agreed with Zhao et al (2018) who demonstrated that persistent expression of CTLA-4 on tumors contributed to the progression of both hematological and solid tumors, which produced inhibitory signals to weaken the immune response.

On the other hand, previous studies found that patients with positive tumor CTLA 4 expression had a better prognosis in NSCLC and gastric cancer (Kim et al., 2016). Moreover, other studies demonstrated that there is no association between CTLA4 expression in tumor cells and any of the clinicopathological parameters either in breast carcinoma or esophageal squamous cell carcinoma (Lan et al., 2018; Zhang et al., 2019).

This discrepancy whether CTLA-4 expression in tumor cells has a good prognostic impact or a bad impact is due to much less data known about its expression and function in tumor cells (Salvi et al., 2012). Moreover, there is increasing evidence that ligands of the immune checkpoint pathways could also trigger a receptor independent signal inside the cells in which they are expressed and that these signals could be different depending on the specific cell types. Therefore, it is still crucial to identify biomarkers that could predict these phenomena and to develop novel preclinical models suitable to investigate the underlining molecular mechanisms (Lecis et al., 2019)

Regarding CTLA 4 expression in lymphocytes the current study demonstrated significant association with good prognostic parameters as absent metastasis, and a trend of significance with low tumor stage. These results agreed with Hu et al., (2017). These results may be explained as CTLA-4 exerts distinct independent effects during different phases of T cell responses, including setting the threshold for T cell activation, suppression of T cell proliferation, and induction of apoptosis in activated T cells (Denkert et al., 2015). On contrary, these results disagreed with Lan et al., (2018) who reported that there is no association between interstitial CTLA 4 expression and any of the clinicopathological parameters and also disagreed with Chang et al., (2017) who reported that CTLA-4 lymphocyte expression showed an association with the presence of vascular emboli. This discrepancy may be explained by Sun et al., (2017) who reported that higher Tregs levels showed an association with poor tumor differentiation and metastasis. 

Furthermore, There is a direct association between positive VDR expression in tumor cells and positive and high CTLA4 expression in lymphocytes and these come in line with Sheikh et al., (2018) who observed that the stimulation of CD4+ T cells with vitamin D suppresses proliferation capacity; enhanced the expression of PD1, PD-L1, and CTLA-4 inhibitory markers on CD4+ T and these results explained by Jeffery et al., (2009) who observed that stimulation of CD4+ CD25− T cells in the presence of calcitriol inhibits production of pro-inflammatory cytokines, induced high levels of CTLA-4 and FoxP3, but does not substantially affect T cell division. 

## Author Contribution Statement

None.
